# GEN1 promotes Holliday junction resolution by a coordinated nick and counter-nick mechanism

**DOI:** 10.1093/nar/gkv1207

**Published:** 2015-11-17

**Authors:** Ying Wai Chan, Stephen West

**Affiliations:** Francis Crick Institute, Clare Hall Laboratories, South Mimms, Herts EN6 3LD, UK

## Abstract

Holliday junctions (HJs) that physically link sister chromatids or homologous chromosomes are formed as intermediates during DNA repair by homologous recombination. Persistent recombination intermediates are acted upon by structure-selective endonucleases that are required for proper chromosome segregation at mitosis. Here, we have purified full-length human GEN1 protein and show that it promotes Holliday junction resolution by a mechanism that is analogous to that exhibited by the prototypic HJ resolvase *E. coli* RuvC. We find that GEN1 cleaves HJs by a nick and counter-nick mechanism involving dual co-ordinated incisions that lead to the formation of ligatable nicked duplex products. As observed with RuvC, cleavage of the first strand is rate limiting, while second strand cleavage is rapid. In contrast to RuvC, however, GEN1 is largely monomeric in solution, but dimerizes on the HJ. Using HJs containing non-cleavable phosphorothioate-containing linkages in one strand, we show that the two incisions can be uncoupled and that the first nick occurs upon GEN1 dimerization at the junction. These results indicate that the mechanism of HJ resolution is largely conserved from bacteria to man, despite a lack of sequence homology between the resolvases.

## INTRODUCTION

Homologous recombination plays an important role in DNA strand break repair. Individuals carrying mutations that affect the efficiency of recombinational repair, such as those carrying *BRCA1* or *BRCA2* mutations, are predisposed to cancer (1). In somatic cells, recombination usually occurs between sister chromatids, although interactions between homologous chromosomes are also observed. Intermediates of recombination often contain structures in which the two recombining DNAs are covalently interlinked by four-way structures known as Holliday junctions (2,3). These structures need to be removed prior to chromosome segregation.

Eukaryotic cells possess two mechanisms for HJ processing: the first is catalyzed by the BTR complex (BLM helicase-Topoisomerase IIIα-RMI1-RMI2) and is known as HJ dissolution, whereas the second involves structure-selective endonucleases such as MUS81-EME1 and GEN1 (4,5). Dissolution leads exclusively to the formation of non-crossovers (NCOs), whereas nucleolytic resolution leads to the formation of both crossovers (COs) and NCOs. Since NCOs are preferred in mitotic cells to prevent loss of heterozygosity (6,7), the actions of the HJ resolving nucleases are restrained until late in the cell cycle where they serve to ensure proper chromosome segregation (8–14).

The structure-selective endonucleases that cut Holliday junctions are usually referred to as HJ resolvases, and have been identified in various organisms, including bacteriophage, bacteria, yeast, archaea and humans (15). The prototypic HJ resolvase, *E. coli* RuvC, a homodimeric protein, binds and cleaves HJs specifically (16–23). Resolution occurs by the introduction of a pair of symmetrically-related nicks in the two strands that lie diametrically opposed across the junction, producing nicked linear duplexes that can be readily repaired by DNA ligase (18). Bilateral cleavage occurs within the lifetime of the protein–HJ complex, with the first incision being rate limiting and the second incision rapid (24). This mechanism of HJ cleavage is analogous to that mediated by other resolvases such as bacteriophage T4 endonuclease VII, T7 endonuclease I, the yeast mitochondrial resolvase Cce1, the plant (*Arabidopsis thaliana*) resolvases *At*GEN1 and *At*SEND1 and the thermophilic (*Chaetomium thermophilum*) resolvase *Ct*GEN1 (24–30). Remarkably, however, these HJ resolvases show little sequence homology, suggesting that convergent evolution processes have led to the adoption of a common mechanism for HJ resolution (31,32).

Higher eukaryotes possess several structure-selective endonucleases that cleave HJs. These include MUS81-EME1 (33,34), SLX1-SLX4 (35–38) and GEN1 (39,40). MUS81-EME1 preferentially cleaves 3′-flaps, replication forks and nicked HJs, but displays only a very weak activity towards HJs (12,34). Unlike canonical resolvases, MUS81 induces asymmetric nicks across the junction, leading to non-ligatable product formation (34,41–43). By comparison, SLX1-SLX4 is a promiscuous endonuclease that cuts 5′- and 3′-flaps, replication forks and HJs (12). Cleavage of the two strands of a HJ by SLX1-SLX4 displays little coordination. However, the combined actions of SLX1-SLX4 and MUS81-EME1, which are known to interact at the prometaphase stage of the cell cycle, allows coordination of their active sites, such that SLX1-SLX4 induces the rate-limiting first incision and MUS81-EME1 mediates the second incision (12). In agreement with these observations, MUS81-EME1 and SLX1-SLX4 act in the same HJ processing pathway in human cells (12,44–46). This pathway is distinct from that involving GEN1, which functions after the breakdown of the nuclear envelope at mitotic entry (47).

GEN1 is a member of Rad2/XPG nuclease family. Members of this family contain an N-terminal (N) and an internal (I) XPG nuclease domain, a helix-hairpin-helix (HhH) domain that is important for DNA binding, and a large C-terminal tail that is predicted to be disordered (39,40). This disordered tail has made purification of the full-length protein problematic, and all previous biochemical analyses of GEN1 have been restricted to the use of recombinant N-terminal fragments (human GEN1^1–^^527^ or *Ct*GEN1^1^^–487^) (30,40).

In the work described here, we have purified full-length human GEN1 protein (908 amino acids), and provide a detailed analysis of its substrate specificity and the mechanism by which it resolves HJs. We find that the protein promotes HJ resolution by a RuvC-like mechanism. We show that the two incisions occur independently and yet near-simultaneously, and importantly that the initial rate-limiting incision occurs upon GEN1 dimerization on the junction. Together, these properties of GEN1 ensure that resolution occurs within the lifetime of the GEN1-HJ complex.

## MATERIALS AND METHODS

### Plasmids for protein expression

The GEN1 and GEN1^1^^–527^ coding sequences were generated by PCR from a human cDNA clone (Origene) and shuttled into pDONR221 by Gateway recombination (Life Technologies). A FLAG tag sequence (AGDYKDDDDK) was added into the C-terminus of each protein. GEN1^E134A, E136A^ (GEN1^EEAA^) was generated using QuikChange Lightning Multi Site-Directed Mutagenesis Kit (Agilent). GEN1, GEN1^1^^–527^ and GEN1^EEAA^ were then shuttled into pYES-DEST52 by Gateway recombination (Life Technologies) for expression in *S. cerevisiae*.

### Protein purification

Plasmids expressing C-terminally FLAG-His-tagged GEN1, GEN1^1^^–527^ or GEN1^EEAA^ were transformed into *S. cerevisiae* W303 strain (pep4Δ::KanMX). Cells were grown exponentially in SC–Ura media at 30°C. Protein expression from the GAL1 promoter was induced by addition of 2% galactose to cultures at OD_650_ ∼1.2. Cells were harvested, washed and disrupted in a freezer mill. The powder was resuspended in lysis buffer (40 mM Tris-HCl, pH 7.5, 500 mM NaCl, 10% glycerol, 2 mM EDTA, 0.1% NP-40, 1 mM DTT and protease inhibitors), cleared by ultracentrifugation at 40 000 rpm for 40 min using Ti45 rotor (Beckman Coulter), and incubated with anti-FLAG M2 agarose beads (Sigma-Aldrich) for 1 h at 4°C. The beads were extensively washed in lysis buffer and washed in ATP buffer (40 mM Tris-HCl, pH 7.5, 500 mM NaCl, 10% glycerol, 0.1% NP-40, 1 mM DTT, 1 mM ATP and 3 mM MgCl_2_). Proteins were eluted with three column volumes of M2 elution buffer (lysis buffer without EDTA, supplemented with 0.5 mg/ml 3xFLAG peptide and 10 mM imidazole). The FLAG eluate was then incubated with Ni-NTA agarose beads (Qiagen) for 1 h at 4°C, and the beads were extensively washed in lysis buffer without EDTA, supplemented with 10 mM imidazole. Finally, purified proteins were eluted with 300 mM imidazole in lysis buffer, and dialyzed against 40 mM Tris-HCl, pH 7.5, 250 mM NaCl, 10% glycerol, 0.1 mM EDTA, 0.05% NP-40 and 1 mM DTT for storage in aliquots at −80°C. Protein concentrations were determined using the Bradford assay (BioRad) and on Instant Blue (Expedeon)-stained SDS-PAGE gels using BSA as the standard. Yen1 was purified as described (13).

### Nuclease assays

The 5′-^32^P-end-labeled synthetic DNA substrates were prepared as described (48), using the oligonucleotides listed in Supplementary Table S1. The oligonucleotide X0–3, containing a phosphorothioate linkage (SP linkage) in which one of the non-bridging oxygens of the phosphodiester bond between 31 and 32 nt was replaced by sulfur (5′-TCsAT-3′), was obtained from Sigma-Aldrich. Following annealing, unlabeled DNAs were purified by native 12% PAGE and visualized by UV-shadowing. For HJ cleavage assays, X0 was used unless stated otherwise. Nuclease assays were carried out in 10 μl cleavage buffer (50 mM Tris-HCl pH 7.5, 1 mM MgCl_2_ and 1 mM DTT) containing unlabeled DNA spiked with ∼1 nM of the same 5′-^32^P-end-labeled DNA. After incubation at 37°C, DNA products were deproteinized by addition of 2.5 μl of stop buffer (100 mM Tris-HCl pH 7.5, 50 mM EDTA, 2.5% SDS and 10 mg/ml proteinase K) and incubation for 15 min at 37°C. The radiolabeled products were then analyzed by PAGE through 10% neutral or 12% denaturing gels, and analyzed by autoradiography or by phosphorimaging using a Typhoon scanner and ImageQuant software (GE Healthcare).

Cruciform extrusion of plasmid pIRbke8^mut^ (40) was stimulated by incubation for 90 min at 37°C in 50 mM Tris-HCl pH 7.5, 50 mM NaCl and 0.1 mM EDTA. Digestion with EcoRI was used to determine the efficiency of cruciform extrusion. Cleavage reactions were carried out at 37°C. The products were analyzed by 0.8% agarose gel electrophoresis, stained with SYBR Gold (Roche) and imaged with a Gel Doc XR+ System (Bio-Rad). Reaction products were quantified using Image Lab software (BioRad) and normalized to reflect the amount of cruciform-extruded substrate (i.e. the amount of plasmid refractory to cleavage by EcoRI).

### Hydrodynamic analyses

Size exclusion chromatography was carried out at 4°C on a Superdex 200 PC 3.2/30 column (GE Healthcare) equilibrated with 40 mM Tris-HCl, pH 7.5, 250 mM NaCl, 10% glycerol, 0.1 mM EDTA, 0.05% NP-40 and 1 mM DTT. GEN1 (50 μl, ∼5 μg) was applied to the column and 50 μl fractions were collected. Glycerol gradient sedimentation was performed using a 15–35% (v/v) 4 ml glycerol density gradient in the same buffer. GEN1 (150 μl, ∼15 μg) was loaded and ultracentrifugation was carried out in a SW55Ti swinging bucket rotor at 42,000 rpm for 16 h at 4°C (Optima LE- 80K Ultracentrifuge, Beckman Coulter) and 140 μl fractions were collected. In both experiments, GEN1 was detected by SDS-PAGE followed by western blotting, and fractions (1 μl) we assayed for HJ resolution. Bio-Rad gel filtration standards (thyroglobulin, γ-globulin, ovalbumin and myoglobin) were used as markers.

### Electrophoretic mobility shift assays (EMSA)

Electrophoretic mobility shift assays (EMSA) were carried out for 15 min on ice by mixing GEN1 or GEN1^1^–^527^ with unlabeled DNA (5 nM) supplemented with ∼0.5 nM of 5′-^32^P-end labeled DNA in 10 μl of binding buffer (50 mM Tris-HCl pH 7.5, 125 mM NaCl, 5 mM EDTA, 1 mM DTT, 100 μg/ml BSA, 6% glycerol, with or without 5 ng/μl poly[dI-dC]). Complexes were separated by 5% neutral PAGE in 0.5x TBE buffer.

## RESULTS

### Specificity of GEN1

**Figure 1.**
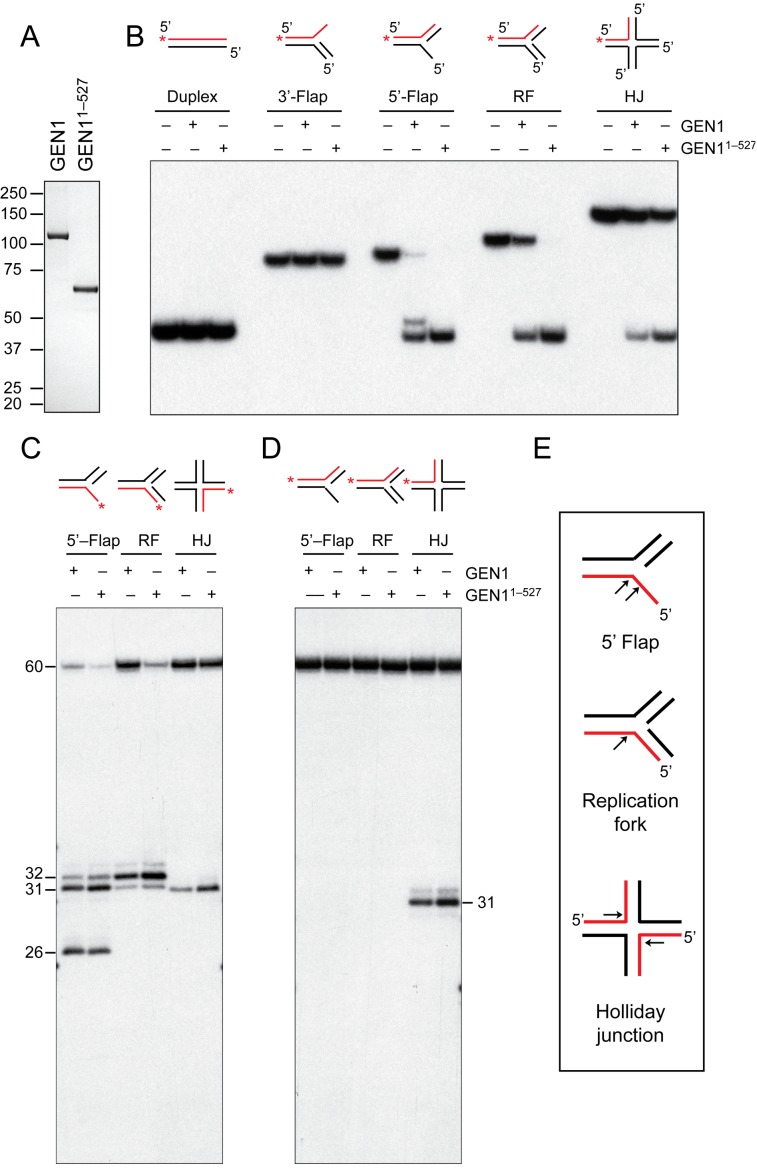
DNA cleavage specificity of GEN1 and GEN1^1–^^527^. (**A**) Purified GEN1 and GEN1^1^^–527^ were analyzed by SDS-PAGE and stained with Instant Blue. Size markers are indicated. (**B**) The indicated DNA substrates (20 nM), 5′-^32^P-end-labeled in strand 1 (red end-labels are indicated with asterisks), were incubated with GEN1 or GEN1^1^^–527^ (5 nM) for 10 min. Products were analyzed by neutral PAGE and visualized by autoradiography. (**C**) and (**D**) As (B), except the DNA substrates, 5′-^32^P end-labeled in either strand 3 (C) or strand 1 (D), were incubated with GEN1 or GEN1^1^^–527^, and the products were analyzed by denaturing PAGE. 5′-^32^P-end-labeled oligos of defined length (25-nt, 30-nt and 35-nt) were used as markers. (**E**) Schematic representation of the cleavage sites (black allows) introduced into the 5′-flap, RF and HJ substrates by GEN1 or GEN1^1^^–527^.

Full-length human GEN1 protein was expressed in yeast using a GAL1 expression system and purified to homogeneity (Figure 1A). For comparative purposes, the previously characterized GEN1^1^^–527^ truncation was purified using the same expression system. The substrate specificities of both proteins were compared using a series of 5′-^32^P-end-labeled linear and branched DNAs produced by annealing the appropriate complementary oligonucleotides. These included linear duplex, 3′-flap, 5′-flap, replication fork (RF) and the immobile Holliday junction X0. In each case, one DNA strand was 5′-^32^P-end-labeled. We found that GEN1 and GEN1^1^^–527^ both cleaved 5′-flap, RF and HJ DNA, as indicated by the appearance of faster-migrating products in neutral PAGE (Figure 1B). In general, we found that GEN1^1^^–527^ was more active than the full-length protein. This difference in activity was not due to phosphorylation in the C-terminus of the protein, as λ phosphatase treatment did not alter their activities (Supplementary Figure S1A). In contrast, and consistent with previous studies (13,14), phosphatase treatment enhanced the activity of Yen1 protein, also purified from yeast. Neither GEN1 nor GEN1^1^^–527^ exhibited any detectable activity with linear duplex and 3′-flap DNAs. GEN1 was free of exonuclease activity as determined using linear duplex and single stranded DNAs (Supplementary Figure S1B).

The sites of incision were determined by denaturing PAGE (Figure 1C and D; see also Figure 1E for the summary). GEN1 and GEN1^1^^–527^ acted upon the 5′-flap substrate to remove the single-stranded flap strand by incision at sites located 1-nucleotide (nt) or 2-nt to the 3′ side of the branch point, giving rise to 31 and 32-nt long fragments on a denaturing gel (Figure 1C, and summarized in Supplementary Figure S2A). We also observed a 26-nt fragment consistent with cleavage 4-nt to the 5′-side of the branch point. Full length GEN1 produced more of this 26-nt fragment than GEN1^1^^–527^, as a consequence of the reduced overall level of cleavage. Cleavage at this distal site removed only part of the 5′-flap, and gives rise to the upper product band on the neutral gel (Figure 1B, and Supplementary Figure S1C). The primary cleavage site on the RF was located 2-nt to the 3′-side of the branch point on the lagging strand template (Figure 1C and D, and Supplementary Figure S2B), indicating that the RF is processed in a similar manner to the 5′-flap.

GEN1 was found to cleave immobile (X0) or mobile (X26) Holliday junctions (Supplementary Figure S1D), although again the efficiency of cleavage by the full-length protein was 2-fold reduced compared with GEN1^1^^–527^ (Supplementary Figure S1E). Cleavage site mapping showed that the incisions in X0 were diametrically opposed at sites located 1-nt to the 3′-side of the junction point (Figure 1C and D, and Supplementary Figures S1F and S2C). As observed previously with GEN1^1^^–527^ (39), resolution occurred in a single orientation (by cleavage of strands 1 and 3), suggesting that this pair of strands adopts the configuration of the continuous strands in the anti-parallel stacked-X structure (40).

**Figure 2.**
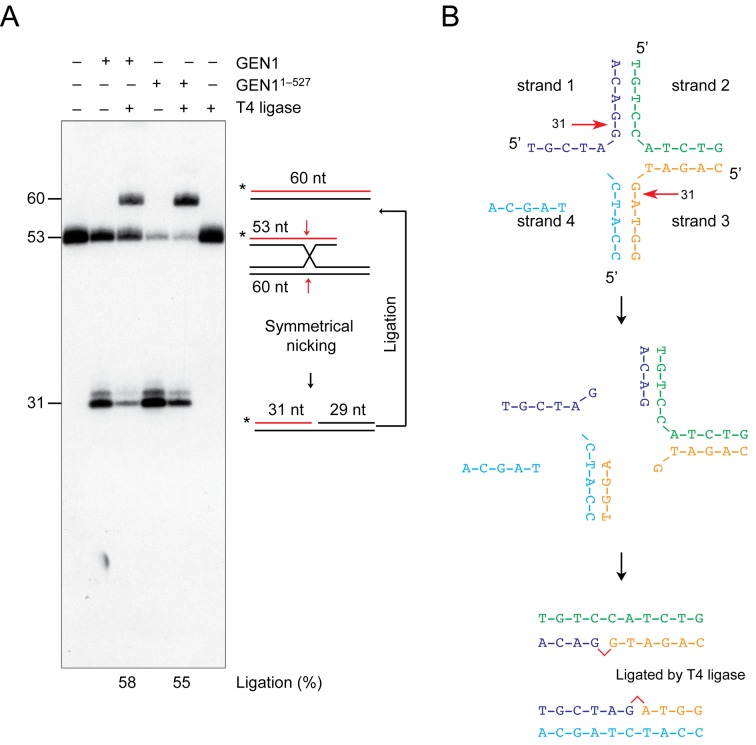
Religation of the resolution products. (**A**) The asymmetric HJ X1-T (∼1 nM) was incubated with GEN1 or GEN1^1^^–527^ (5 nM) for 10 min. Reactions were then supplemented with ligation buffer and T4 DNA ligase (400 U, NEB) and incubated for 1 h at room temperature. Products were analyzed by denaturing PAGE and ligation products were quantified. The schematic indicates how symmetrical cleavage allows nick ligation and converts the radiolabeled 53-nt strand into the 60-nt product. (**B**) Schematic diagram indicating the cleavage and nick-ligation reaction.

To determine whether GEN1 gives rise to ligatable products, we used an asymmetric junction (X1-T) in which the length of one, ^32^P-end-labeled, arm was reduced to 53 bp. When X1-T was incubated with GEN1 or GEN1^1^^–527^, followed by addition of T4 DNA ligase, we observed that greater than 50% of the cleavage products were religated giving rise to a 60-nt long ^32^P-end-labeled strand (Figure 2A). A schematic of the ligation reaction is shown in Figure 2B. These results confirm that cleavage occurs with perfect symmetry at the junction point.

### GEN1 is monomeric in solution

**Figure 3.**
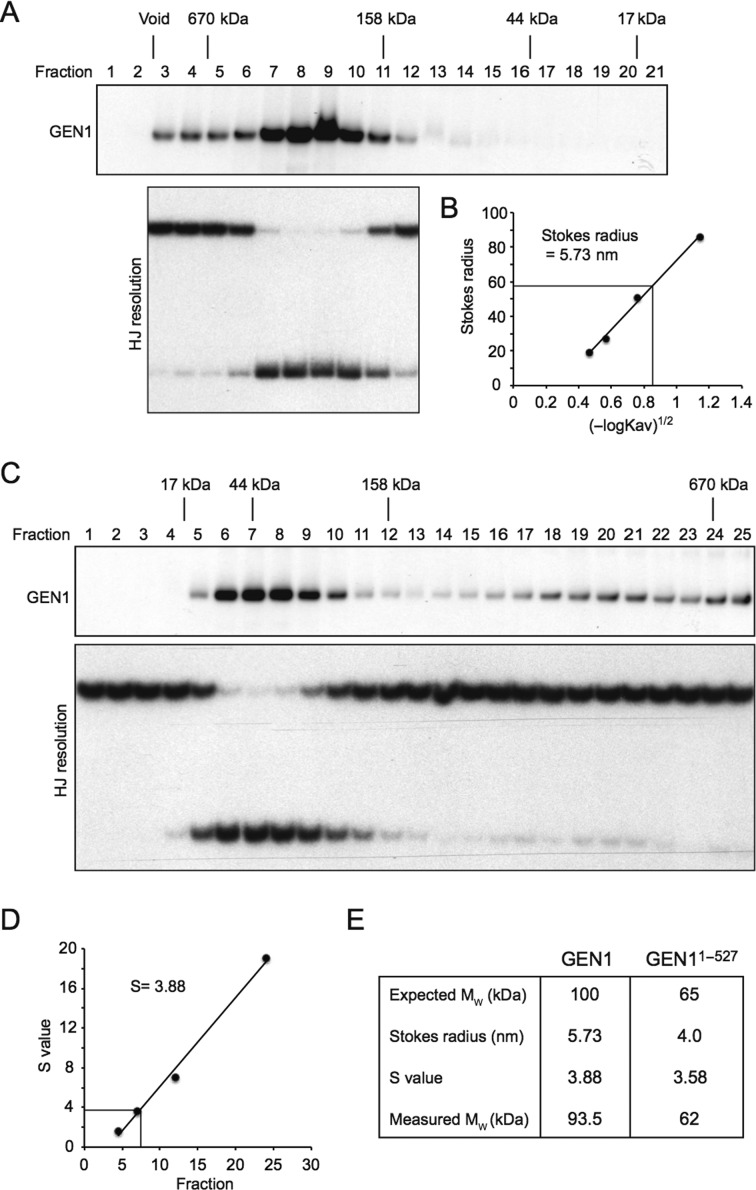
Hydrodynamic analysis of GEN1. (**A**) GEN1 was analyzed by size exclusion chromatography followed by western blotting (upper panel). Molecular mass standards are indicated. Fractions were also assayed for HJ resolution activity using neutral PAGE (lower panel). (**B**) Stokes radius of GEN1, as determined in (A). (**C**) Glycerol gradient sedimentation of GEN1. GEN1 and HJ resolution activity was detected as in (A). (**D**) S-value of GEN1, as determined in (C). (**E**) Summary of the parameters of GEN1 and GEN1^1^^–527^.

The RuvC homodimer has two symmetrically related active sites that promote HJ resolution (20,23). To determine the solution state of GEN1, the protein was analyzed by size exclusion chromatography (Figure 3A and B) and glycerol gradient sedimentation (Figure 3C and D). In each case, purified GEN1 eluted as a single peak, with a Stokes radius of 58 Å and a Svedberg coefficient (S-value) of 3.88. The calculated native molecular weight (M_W_) of GEN1 is 93.5 kDa (Figure 3E). These results indicate that GEN1, like GEN1^1^^–527^ (40), is primarily monomeric in solution.

### Dimerization of GEN1 on the junction promotes coordinated bilateral cleavage

**Figure 4.**
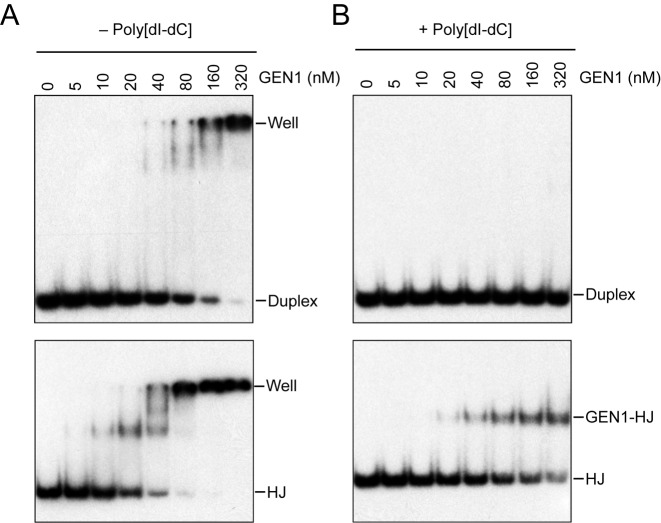
EMSA of GEN1-HJ complexes. (**A**) and (**B**) GEN1 was incubated with linear duplex (upper panels) or HJ (lower panels) DNA in the absence (A) or presence (B) poly[dI-dC], and the complexes were analyzed by neutral PAGE. The specific GEN1-HJ complex is indicated.

The association of GEN1 with HJ DNA was visualized by EMSA. In the absence of a competitor DNA, we found that GEN1 readily associated to both linear duplex and HJ DNA (Figure 4A). The weak non-specific binding to duplex DNA, however, could be competed away by the addition of excess competitor poly[dI-dC] (Figure 4B). Under these conditions, we observed a defined GEN1-HJ complex HJ (Figure 4B), showing that GEN1 forms a stable and specific complex with HJ DNA. GEN1^1^^–527^ displayed a higher affinity to HJ DNA compared with the full-length protein (compare Figure 4 and Supplementary Figure S3), providing an explanation for the higher activity exhibited by GEN1^1^^–527^.

**Figure 5.**
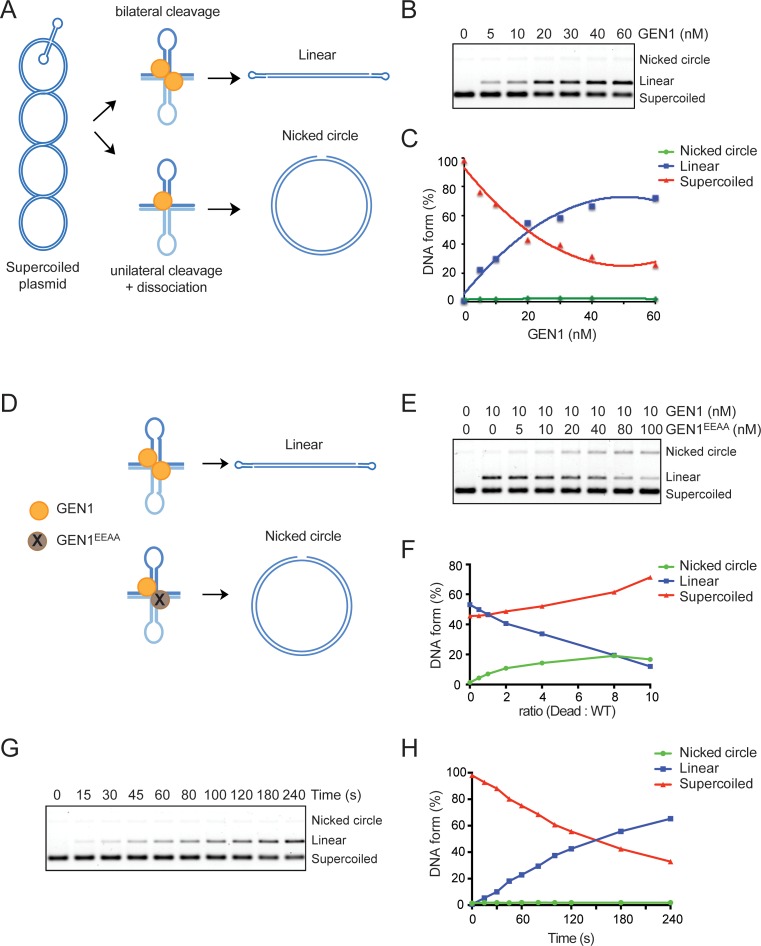
HJ resolution by GEN1 involves coordinated dual incision. (**A**) Schematic representation of the cruciform cleavage assay used to determine whether GEN1 introduces coordinated dual incisions within the lifetime of the protein–HJ complex. (**B**) Plasmid pIRbke8^mut^ (1 nM) was incubated with GEN1 for 5 min. Products were analyzed by agarose gel electrophoresis. (**C**) Quantification of the DNA products, as determined in (B). (**D**) Schematic representation indicating the formation of nicked circular DNA by the cooperative action of catalytically inactive GEN1 (GEN1^EEAA^) and wild-type GEN1. (**E**) Plasmid pIRbke8^mut^ (0.5 nM) was incubated with the indicated concentrations of GEN1 and GEN1^EEAA^ for 5 min. (**F**) Quantification of the DNA products, as determined in (E). (**G**) Plasmid pIRbke8^mut^ (1 nM) was incubated with GEN1 (20 nM) for the indicated times. (**H**) Quantification of the DNA, as determined in (G).

To determine whether GEN1 dimerizes upon binding to the HJ, providing two active sites for dual incision, we made use of the supercoiled plasmid pIRbke8^mut^. This plasmid contains an inverted repeat sequence that extrudes to form a cruciform structure (40,49). Coordinated bilateral cleavage of the cruciform results in the formation of a linear product, whereas unilateral cleavage followed by enzyme–DNA dissociation generates a nicked circular plasmid. The nicking of the plasmid leads to the loss of superhelical stress, resulting in cruciform reabsorption and the subsequent inability to serve as a substrate for resolution (Figure 5A). This substrate therefore provides a useful tool to demonstrate coordinated cleavage by a dimeric HJ resolvase. We found that GEN1 cleaved pIRbke8^mut^ to exclusively form linear products (Figure 5B and C), indicating that GEN1 mediates the dual incision of the junction within the lifetime of the enzyme–DNA complex.

To define the mechanism of HJ resolution by GEN1 in greater detail, we generated a catalytically dead version of GEN1, GEN1^EEAA^ (39), by mutating the active site residues E134 and E136 to alanine (Supplementary Figure S4A). When mixed with wild-type GEN1, we observed reduced levels of linear product, and instead saw an increasing fraction of nicked circular DNA in the cruciform cleavage assay (Figure 5D–F). Reasoning that GEN1-GEN1^EEAA^ heterodimers are responsible for generating the nicked circular products (Figure 5D), these results show that it is possible to uncouple the two (normally coordinated) cleavage events. The results also show that one active subunit of the dimer can still function normally when associated with a catalytically inactive partner. The generation of nicked circular DNA in these experiments was not due to any contaminating nuclease activity in the preparation of GEN1^EEAA^, as this protein alone exhibited no activity with oligo-based synthetic substrates (5′-flap, RF and HJ) or the cruciform plasmid (Supplementary Figure S4B and C). We therefore conclude that the coordination of dual incision is achieved by GEN1 dimerization.

### Near-simultaneous cleavage by acceleration of the second incision

The results described above indicate that the two active sites of the GEN1 dimer mediate a coordinated nick and counter-nick reaction. This was confirmed in time course experiments with pIRbke8^mut^ which showed that linear duplex DNA was the only cleavage product, even at the shortest time points (Figure 5G and H), and indicated that the dual incisions occurs near-simultaneously. Our results therefore raise the possibility that second-strand cleavage occurs at an increased rate compared with the first strand, as previously observed with RuvC (24). Indeed, with RuvC, it was shown that the presence of a strand break at the point of strand exchange accelerates cleavage of the opposing strand due to the increased flexibility of the junction (24). To determine whether this was also true for GEN1, we compared the rate of cleavage of intact versus nicked HJ by GEN1 and found that the nicked HJ was cleaved at a faster rate than the intact HJ (Supplementary Figure S4D and E). These results show that the first nick is rate limiting and that the GEN1, like RuvC, exhibits accelerated second-strand incision.

### The first strand incision occurs upon GEN1 dimerization

**Figure 6.**
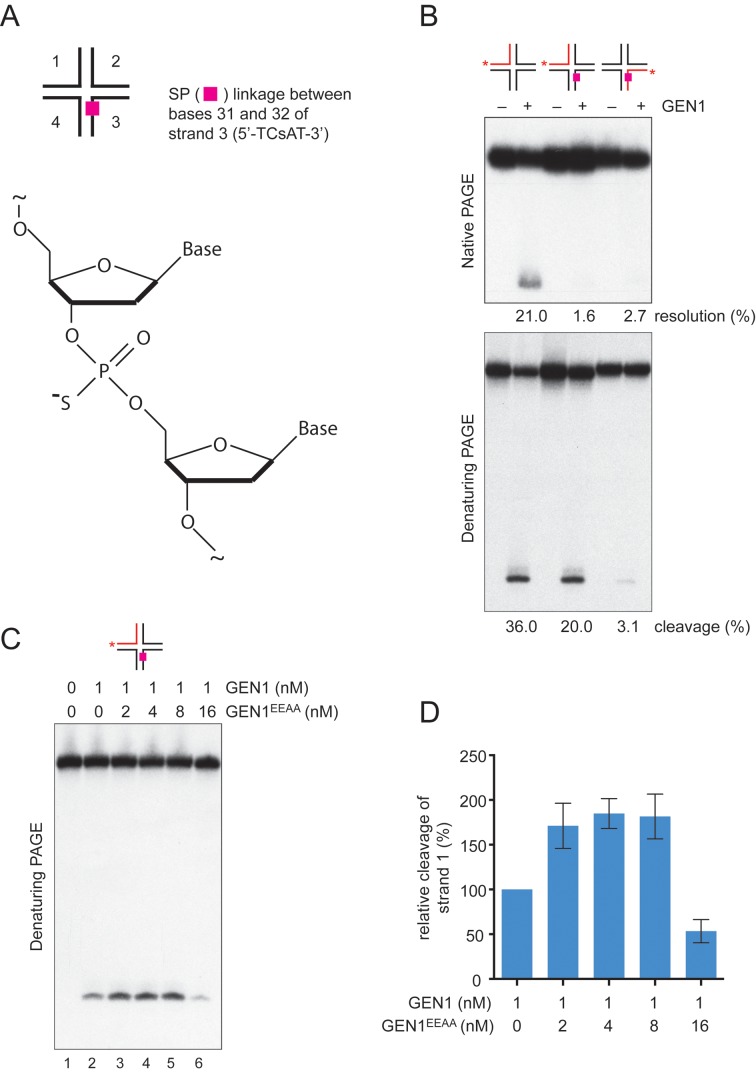
Uncoupling of the dual incisions that promote HJ cleavage. (**A**) Incorporation of a phosphorothioate (SP) linkage between nucleotides 31 and 32 in strand 3 of the HJ (5′-TCsAT-3′). The other strands were unmodified. (**B**) HJ DNA (1 nM) with or without the SP linkage, as indicated, was incubated with GEN1 (1 nM) for 5 min. Products were analyzed by neutral (upper panel) and denaturing (lower panel) PAGE. DNA was labeled in the indicated strands (red). Cleavage was quantified as shown. (**C**) HJ DNA (1 nM) containing an SP link was incubated with GEN1 (1 nM) and the indicated concentrations of GEN1^EEAA^ for 2 min. Products were analyzed by denaturing PAGE. (**D**) Quantification of the reactions shown in (C). Cleavage by GEN1 alone is represented as 100%. The data represent the mean ±SD of three independent experiments.

Next, we wanted to determine whether the near-simultaneous nature of the dual incision reaction was dependent upon the dimerization of GEN1. We therefore constructed a version of X0 HJ in which the cleavage site in strand 3 was made resistant to cleavage by incorporation of a hydrolysis-resistant phosphorothioate (SP) linkage between 31-nt and 32-nt (i.e. 5′-TCsAT-3′) (Figure 6A). When the SP-modified HJ was incubated with GEN1, we observed that the DNA was refractory to resolution by GEN1, as determined by native PAGE (Figure 6B, upper panel). However, when the reaction products were analyzed by denaturing PAGE, we found that the unmodified strand opposite that containing the SP linkage was cleaved (Figure 6B, lower panel). These results show that SP-modified HJ DNA serves as a substrate for GEN1, and that the two incisions now become uncoupled.

If the first incision is dependent upon dimerization, we suggest that it might be possible to enhance first strand cleavage by favoring dimer formation. We therefore incubated the SP-modified HJ (5′-^32^P-end-labeled in the unmodified strand 1) with wild-type GEN1 and increasing amounts of GEN1^EEAA^. Strikingly, we found that the presence of the catalytically dead GEN1^EEAA^ mutant enhanced cleavage of strand 1 (Figure 6C, lanes 2–5, and quantified in 6D). Cleavage only became inhibited when GEN1^EEAA^ was in large excess (16:1) over wild-type GEN1, presumably because mutant dimers outcompete GEN1 for junction binding. These results show that the initial rate-limiting incision occurs upon GEN1 dimerization.

These results show that HJ resolution mediated by GEN1 is dependent upon its dimerization by binding to a HJ. This mode of action appears to be different from 5′-flap cleavage, which is thought to be mediated by a single GEN1 monomer. To confirm that this was indeed the case, HJ or 5′-flap DNAs were incubated with GEN1 and increasing amount of catalytically dead GEN1^EEAA^ (Supplementary Figure S5A and B). We found that the cleavage of the 5′-flap was more readily inhibited by GEN1^EEAA^ compared with cleavage of the HJ, consistent with the notion that GEN1 is a monomeric 5′-flap endonuclease.

## DISCUSSION

In this work, we provide the first biochemical characterization of the full-length GEN1 protein. Like other members of Rad2/XPG nuclease family (such as FEN1), GEN1 acts as a monomeric endonuclease that cleaves 5′-flap DNA, but unique to this family, GEN1 mediates the cleavage of Holliday junctions by a mechanism similar to that of the prototypic resolvase RuvC. As such, GEN1 is the only human HJ resolvase that introduces symmetrically related nicks across the junction to produce a pair of ligatable nicked duplexes.

To date, all resolvases, such as bacteriophage T4 endonuclease VII, bacterial RuvC and RusA, yeast mitochondrial Cce1, and thermophilic *Ct*GEN1, interact with HJs as homodimers (20,22,23,26–28,30,50). In contrast, however, we find that GEN1 is monomeric in solution, but importantly, homodimerization with the Holliday junction provides the two active sites necessary for the introduction of dual and coordinated incisions. The dual incisions mediated by GEN1 occur within the lifetime of the enzyme-junction complex and appear to occur near-simultaneously. However, using two different approaches, we found that the two incisions could be uncoupled. Firstly, using cruciform plasmid DNA mixed with wild-type GEN1 and catalytically dead GEN1^EEAA^, we observed the generation of single-nicked products. Secondly, using synthetic substrates in which one of the scissile phosphates was replaced by a hydrolysis-resistant phosphorothioate bond, we found that the opposing strand remained a target for incision. These results show that GEN1 promotes HJ resolution by an ordered nick and counter-nick mechanism.

Furthermore, our analyses uncovered two mechanisms that ensure dual sequential incisions occur within the lifetime of the GEN1-HJ complex. We found that the initial rate-limiting incision occurs upon GEN1 dimerization. The requirement for dimer formation for the first incision prevents non-productive nicking of the HJ when a single GEN1 subunit binds to the junction. One possible explanation for this phenomenon is that dimer formation induces a conformational change that triggers the first cleavage. Consistent with this idea, it has been shown that the binding of a RuvC dimer to the Holliday junction induces junction unfolding (21). We also found, as observed in other resolvases, that the rate of second strand cleavage is accelerated by first strand cleavage. This may be due to the relaxation of stress at the point of the crossover that occurs upon introduction of the first nick. Acceleration of the second strand incision greatly increases the probability that both cleavages occur before dissociation of the enzyme–junction complex. Taken together, the enzymatic properties of GEN1 ensure productive resolution by coordinating the dual incisions within the lifetime of the GEN1-HJ complex.

The biochemical properties of full-length GEN1 described here are similar to those reported previously for the N-terminal GEN1^1^^–527^ truncation. Indeed, we find that GEN1 and GEN1^1^^–527^ exhibit the same substrate specificity and promote cleavage at the same sites. Additionally, both GEN1 and GEN1^1^^–527^ are monomeric in solution, suggesting that the domains necessary for dimerization on the HJ are present in the N-terminal portion of the protein. Our recent work revealed that the C-terminus of GEN1 contains a nuclear export signal (NES) that controls the subcellular localization of the protein (47). In addition to the NES, however, we do not exclude the possibility that there may be posttranslational modifications or interaction motifs in the C-terminus that serve other regulatory functions. Together, these observations indicate that the C-terminus of GEN1 does not affect the catalytic function of the resolvase, but instead is important for regulating the cell cycle stage at which the protein promotes resolution. Since GEN1 is primarily cytoplasmic, it can only act upon DNA following disruption of the nuclear envelope. At this time GEN1 is important for the resolution of any persistent HJs that have escaped the attention of the BTR and SLX-MUS complexes, and so guarantee proper chromosome segregation (47,51). The monomer-dimer transition that GEN1 can undergo, together with its ability to promote the resolution of 5′-flaps and replication fork structures may, however, indicate that the actions of GEN1 at anaphase are not restricted to HJ resolution. Indeed, monomeric GEN1 may be responsible for processing 5′-flaps and unresolved replication forks, while GEN1 dimerizes upon binding to HJs to catalyze their resolution. As such, we suggest that GEN1 functions as a jack-of-all-trades that tidies up DNA in preparation for cytokinesis.

## Supplementary Material

SUPPLEMENTARY DATA

## References

[1] Venkitaraman A.R. (2014). Cancer suppression by the chromosome custodians, BRCA1 and BRCA2. Science.

[2] Holliday R. (1964). A mechanism for gene conversion in fungi. Genet. Res. Camb..

[3] Bzymek M., Thayer N.H., Oh S.D., Kleckner N., Hunter N. (2010). Double Holliday junctions are intermediates of DNA break repair. Nature.

[4] Matos J., West S.C. (2014). Holliday junction resolution: regulation in space and time. DNA Rep..

[5] Sarbajna S., West S.C. (2014). Holliday junction processing enzymes as guardians of genome stability. Trends Biochem. Sci..

[6] Luo G.B., Santoro I.M., McDaniel L.D., Nishijima I., Mills M., Youssoufian H., Vogel H., Schultz R.A., Bradley A. (2000). Cancer predisposition caused by elevated mitotic recombination in Bloom's mice. Nat. Genet..

[7] Wu L., Hickson I.D. (2003). The Bloom's syndrome helicase suppresses crossing over during homologous recombination. Nature.

[8] Matos J., Blanco M.G., Maslen S.L., Skehel J.M., West S.C. (2011). Regulatory control of the resolution of DNA recombination intermediates during meiosis and mitosis. Cell.

[9] Gallo-Fernandez M., Sauger I., Ortiz-Bazan M.A., Vazquez M.V., Tercero J.A. (2012). Cell cycle-dependent regulation of the nuclease activity of Mus81-Eme1/Mms4. Nucleic Acids Res..

[10] Szakal B., Branzei D. (2013). Premature Cdk1/Cdc5/Mus81 pathway activation induces aberrant replication and deleterious crossover. EMBO J..

[11] Matos J., Blanco M.G., West S.C. (2013). Cell cycle kinases coordinate the resolution of recombination intermediates with chromosome segregation. Cell Rep..

[12] Wyatt H.D.M., Sarbajna S., Matos J., West S.C. (2013). Coordinated actions of SLX1-SLX4 and MUS81-EME1 for Holliday junction resolution in human cells. Mol. Cell.

[13] Blanco M.G., Matos J., West S.C. (2014). Dual control of Yen1 nuclease activity and cellular localization by Cdk and Cdc14 prevents genome instability. Mol. Cell.

[14] Eissler C.L., Mazón G., Powers B.L., Savinov S.N., Symington L.S., Hall M.C. (2014). The Cdk/Cdc14 module controls activation of the Yen1 Holliday junction resolvase to promote genome stability. Mol. Cell.

[15] Wyatt H.D.M., West S.C. (2014). Holliday junction resolvases. Cold Spring Harb. Perspect. Biol..

[16] Dunderdale H.J., Benson F.E., Parsons C.A., Sharples G.J., Lloyd R.G., West S.C. (1991). Formation and resolution of recombination intermediates by *E. coli* RecA and RuvC proteins. Nature.

[17] Iwasaki H., Takahagi M., Shiba T., Nakata A., Shinagawa H. (1991). *Escherichia coli* RuvC protein is an endonuclease that resolves the Holliday structure. EMBO J..

[18] Bennett R.J., Dunderdale H.J., West S.C. (1993). Resolution of Holliday junctions by RuvC resolvase: cleavage specificity and DNA distortion. Cell.

[19] Benson F.E., West S.C. (1994). Substrate specificity of the *Escherichia coli* RuvC protein: resolution of 3- and 4-stranded recombination intermediates. J. Biol. Chem..

[20] Ariyoshi M., Vassylyev D.G., Iwasaki H., Nakamura H., Shinagawa H., Morikawa K. (1994). Atomic structure of the RuvC resolvase: a Holliday junction-specific endonuclease from *E. coli*. Cell.

[21] Bennett R.J., West S.C. (1995). Structural analysis of the RuvC-Holliday junction complex reveals an unfolded junction. J. Mol. Biol..

[22] Shah R., Cosstick R., West S.C. (1997). The RuvC dimer resolves Holliday junctions by a dual incision mechanism that involves base-specific contacts. EMBO J..

[23] Gorecka K.M., Komorowska W., Nowotny M. (2013). Crystal structure of RuvC resolvase in complex with Holliday junction substrate. Nucleic Acids Res..

[24] Fogg J.M., Lilley D.M.J. (2000). Ensuring productive resolution by the junction-resolving enzyme RuvC: large enhancement of the second-strand cleavage rate. Biochemistry.

[25] Giraud-Panis M.J.E., Lilley D.M.J. (1997). Near simultaneous DNA cleavage by the subunits of the junction-resolving enzyme T4 endonuclease VII. EMBO J..

[26] Pöhler J.R.G., Giraud-Panis M.J.E., Lilley D.M.J. (1996). T4 endonuclease VII selects and alters the structure of the four-way junction; binding of a resolution-defective mutant enzyme. J. Mol. Biol..

[27] White M.F., Lilley D.M.J. (1996). The structure-selectivity and sequence-preference of the junction-resolving enzyme CCE1 of *Saccharomyces cerevisiae*. J. Mol. Biol..

[28] Parkinson M.J., Lilley D.M.J. (1997). The junction resolving enzyme T7 endonuclease. 1. Quaternary structure and interaction with DNA. J. Mol. Biol..

[29] Bauknecht M., Kobbe D. (2014). *At*GEN1 and *At*SEND1, two paralogs in *Arabidopsis*, possess Holliday junction resolvase activity. Plant Physiol..

[30] Freeman A.D.J., Liu Y., Declais A.C., Gartner A., Lilley D.M.J. (2014). GEN1 from a thermophilic fungus is functionally closely similar to non-eukaryotic junction-resolving enzymes. J. Mol. Biol..

[31] Aravind L., Makarova K.S., Koonin E.V. (2000). Holliday junction resolvases and related nucleases: identification of new families, phyletic distribution and evolutionary trajectories. Nucl. Acids Res..

[32] Lilley D.M.J., White M.F. (2000). Resolving the relationships of resolving enzymes. Proc. Natl. Acad. Sci. U.S.A..

[33] Chen X.B., Melchionna R., Denis C.M., Gaillard P.H.L., Blasina A., Van de Weyer I., Boddy M.N., Russell P., Vialard J., McGowan C.H. (2001). Human MUS81-associated endonuclease cleaves Holliday junctions *in vitro*. Mol. Cell.

[34] Ciccia A., Constantinou A., West S.C. (2003). Identification and characterization of the human MUS81/EME1 endonuclease. J. Biol. Chem..

[35] Andersen S.L., Bergstralh D.T., Kohl K.P., LaRocque J.R., Moore C.B., Sekelsky J. (2009). *Drosophila* MUS312 and the vertebrate ortholog BTBD12 interact with DNA structure-specific endonucleases in DNA repair and recombination. Mol. Cell.

[36] Fekairi S., Scaglione S., Chahwan C., Taylor E.R., Tissier A., Coulon S., Dong M.Q., Ruse C., Yates J.R., Russell P. (2009). Human SLX4 is a Holliday junction resolvase subunit that binds multiple DNA repair/recombination endonucleases. Cell.

[37] Munoz I.M., Hain K., Declais A.C., Gardiner M., Toh G.W., Sanchez-Pulido L., Heuckmann J.M., Toth R., Macartney T., Eppink B. (2009). Coordination of structure-specific nucleases by human SLX4/BTBD12 is required for DNA repair. Mol. Cell.

[38] Svendsen J.M., Smogorzewska A., Sowa M.E., O'Connell B.C., Gygi S.P., Elledge S.J., Harper J.W. (2009). Mammalian BTBD12/SLX4 assembles a Holliday junction resolvase and is required for DNA repair. Cell.

[39] Ip S.C.Y., Rass U., Blanco M.G., Flynn H.R., Skehel J.M., West S.C. (2008). Identification of Holliday junction resolvases from humans and yeast. Nature.

[40] Rass U., Compton S.A., Matos J., Singleton M.R., Ip S.C.Y., Blanco M.G., Griffith J.D., West S.C. (2010). Mechanism of Holliday junction resolution by the human GEN1 protein. Genes Dev..

[41] Constantinou A., Chen X.-B., McGowan C.H., West S.C. (2002). Holliday junction resolution in human cells: Two junction endonucleases with distinct substrate specificities. EMBO J..

[42] Gaillard P.-H.L., Noguchi E., Shanahan P., Russell P. (2003). The endogenous Mus81-Eme1 complex resolves Holliday junctions by a nick and couternick mechanism. Mol. Cell.

[43] Ehmsen K.T., Heyer W.D. (2008). *Saccharomyces cerevisiae* Mus81-Mms4 is a catalytic, DNA structure-selective endonuclease. Nucleic Acids Res..

[44] Wechsler T., Newman S., West S.C. (2011). Aberrant chromosome morphology in human cells defective for Holliday junction resolution. Nature.

[45] Garner E., Kim Y., Lach F.P., Kottemann M.C., Smogorzewska A. (2013). Human GEN1 and the SLX4-associated nucleases MUS81 and SLX1 are essential for the resolution of replication-induced Holliday junctions. Cell Rep..

[46] Castor D., Nair N., Déclais A.C., Lachaud C., Toth R., Macartney T.J., Lilley D.M.J., Arthur J.S., Rouse J. (2013). Cooperative control of Holliday junction resolution and DNA repair by the SLX1 and MUS81-EME1 nucleases. Mol. Cell.

[47] Chan Y.W., West S.C. (2014). Spatial control of the GEN1 Holliday junction resolvase ensures genome stability. Nat. Comms..

[48] Rass U., West S.C., Campbell JL, Modrich P (2006). Synthetic junctions as tools to identify and characterise Holliday junction resolvases. Meth. Enzymol..

[49] Lilley D.M.J., Kemper B. (1984). Cruciform-resolvase interactions in supercoiled DNA. Cell.

[50] Giraud-Panis M.J.E., Lilley D.M.J. (1998). Structural recognition and distortion by the DNA junction-resolving enzyme RusA. J. Mol. Biol..

[51] Sarbajna S., Davies D., West S.C. (2014). Roles of SLX1-SLX4, MUS81-EME1 and GEN1 in avoiding genome instability and mitotic catastrophe. Genes Dev..

