# Supplementary material for: GEN1 promotes Holliday junction resolution by a coordinated nick and counter-nick mechanism
Source: Nucleic Acids Res. 2015 Nov 17;43(22):10882–92. doi: 10.1093/nar/gkv1207 (PMC4678824; doi:10.1093/nar/gkv1207)
Supplement: SUPPLEMENTARY DATA [file supp_43_22_10882__index.html]

GEN1 promotes Holliday junction resolution by a coordinated nick and counter-nick mechanism — SUPPLEMENTARY DATA 

# GEN1 promotes Holliday junction resolution by a coordinated nick and counter-nick mechanism

## SUPPLEMENTARY DATA

- SUPPLEMENTARY DATA
