# Supplementary material for: GEN1 promotes Holliday junction resolution by a coordinated nick and counter-nick mechanism
Source: Nucleic Acids Res. 2015 Nov 17;43(22):10882–92. doi: 10.1093/nar/gkv1207 (PMC4678824; doi:10.1093/nar/gkv1207)
Supplement: SUPPLEMENTARY DATA [file supp_gkv1207_nar-02599-z-2015-File008.pdf]

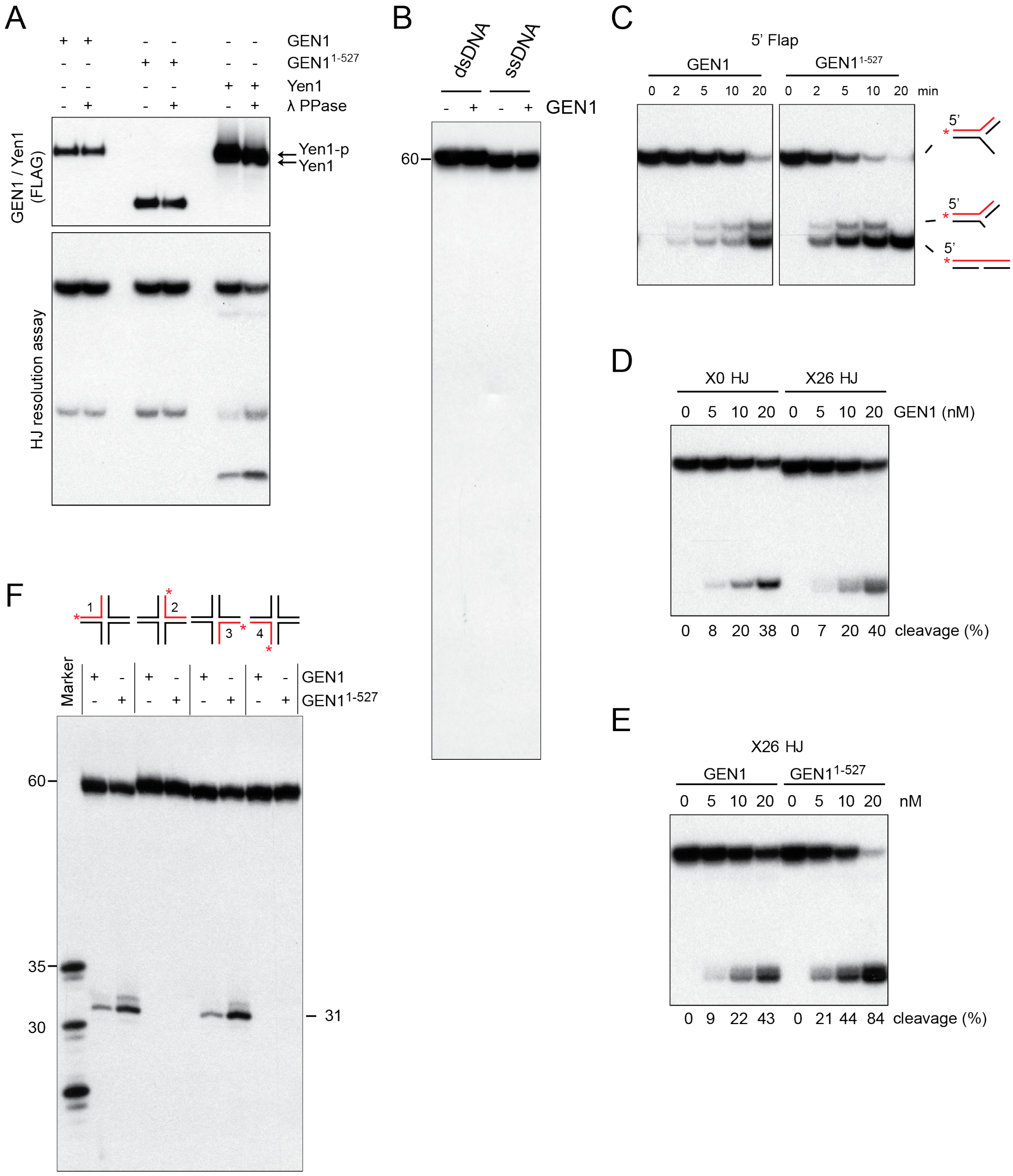

Supplementary Figure S1: HJ cleavage by GEN1.

(A) HJ DNA (~1 nM) was incubated with FLAG-tagged GEN1 (1 nM), GEN1$^{1-527}$ (1 nM) or Yen1 (9 nM) for 5 min. All proteins were preincubated with or without λ-phosphatase (NEB) for 15 min at 30°C, as indicated. Reaction mixtures were analyzed by western blotting with anti-FLAG antibody and the DNA products were analyzed by neutral PAGE. (B) Double and single (poly dT) stranded DNA (~1 nM) were incubated with GEN1 (10 nM) for 15 min. Products were analyzed by denaturing PAGE. (C) 5'-flap DNA (50 nM) was incubated with GEN1 or GEN1$^{1-527}$ (5 nM) for the indicated times, and the reaction products were analyzed by neutral PAGE. The schematics indicate the structures of the 5'-^{32}P-end labeled products. (D) HJs X0 or X26 (50 nM) were incubated with the indicated concentrations of GEN1 for 10 min. Products were analyzed by neutral PAGE. (E) The HJ X26 (50 nM) was incubated with the indicated concentrations of GEN1 or GEN1$^{1-527}$ for 10 min. Products were analyzed by neutral PAGE. (F) HJ DNAs (20 nM), 5'-^{32}P-end-labeled in each of the 4 strands (labels are indicated with asterisks), were incubated with GEN1 or GEN1$^{1-527}$ (5 nM) for 10 min. Products were analyzed by denaturing PAGE.

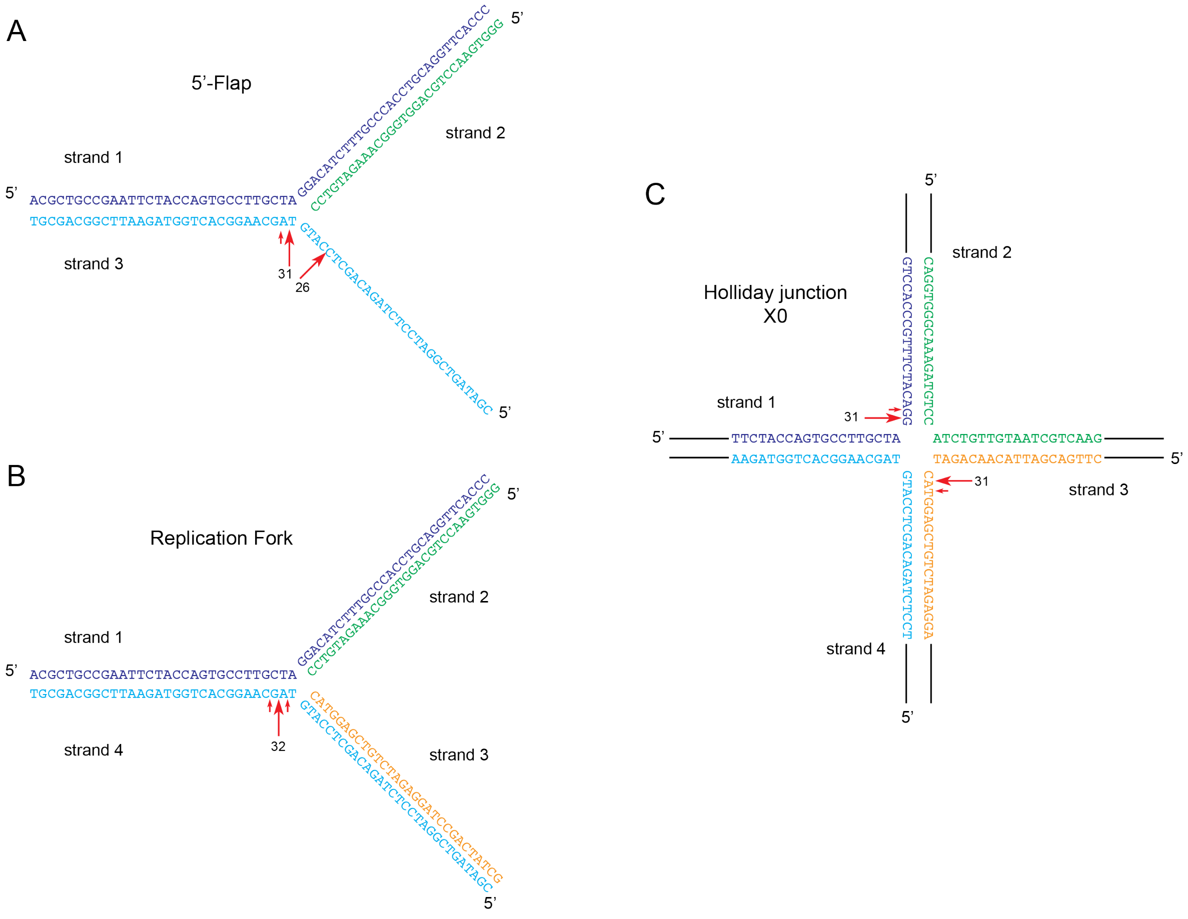

Supplementary Figure S2: Cleavage of 5'-flap, RF and HJ by GEN1.

Mapping of the GEN1 cleavage sites on 5'-flap DNA, replication fork DNA and HJ DNA. Arrow size (red) is proportional to the relative efficiency of nicking at that site.

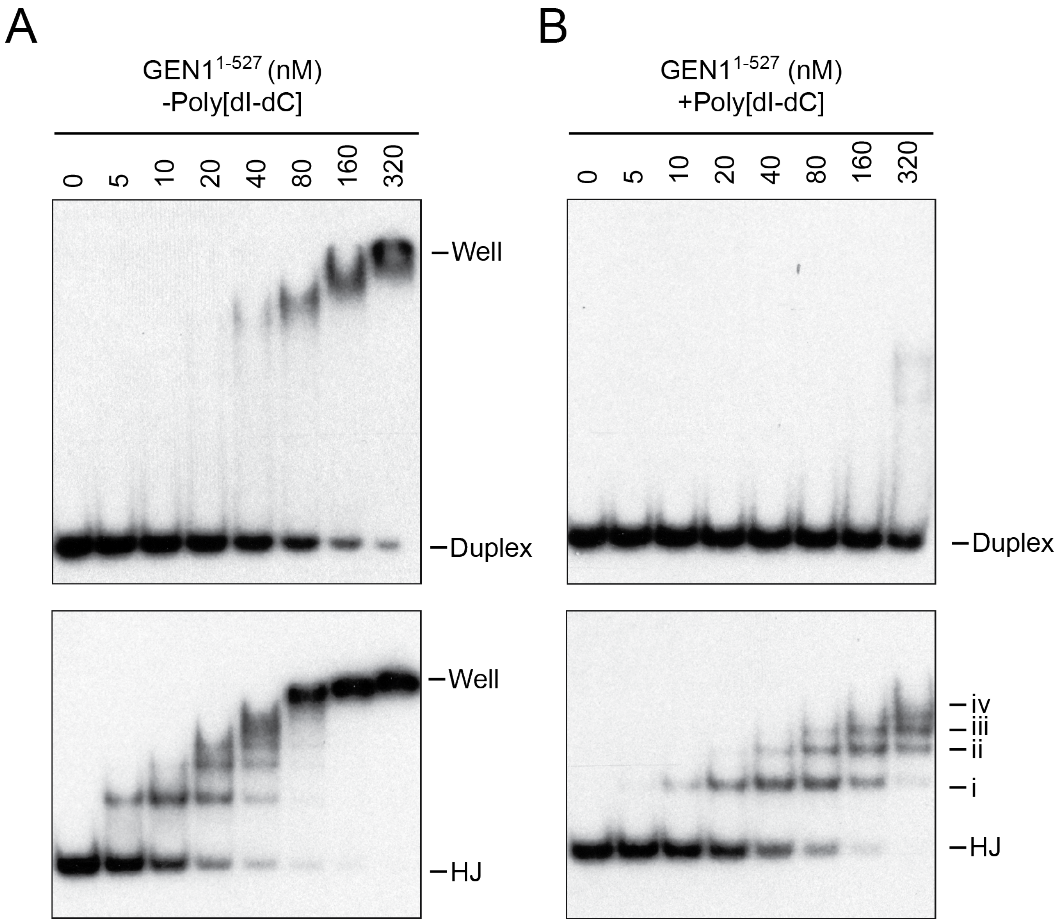

Supplementary Figure S3: EMSA of GEN1$^{1-527}$-HJ complexes.

GEN1$^{1-527}$ was incubated with 5 nM linear duplex (upper panel) or HJ DNA (lower panel) in the absence (A) or presence (B) of poly[dI-dC], and the complexes were analyzed by neutral PAGE. Specific GEN1$^{1-527}$-HJ complexes are indicated (i, ii, iii, iv).

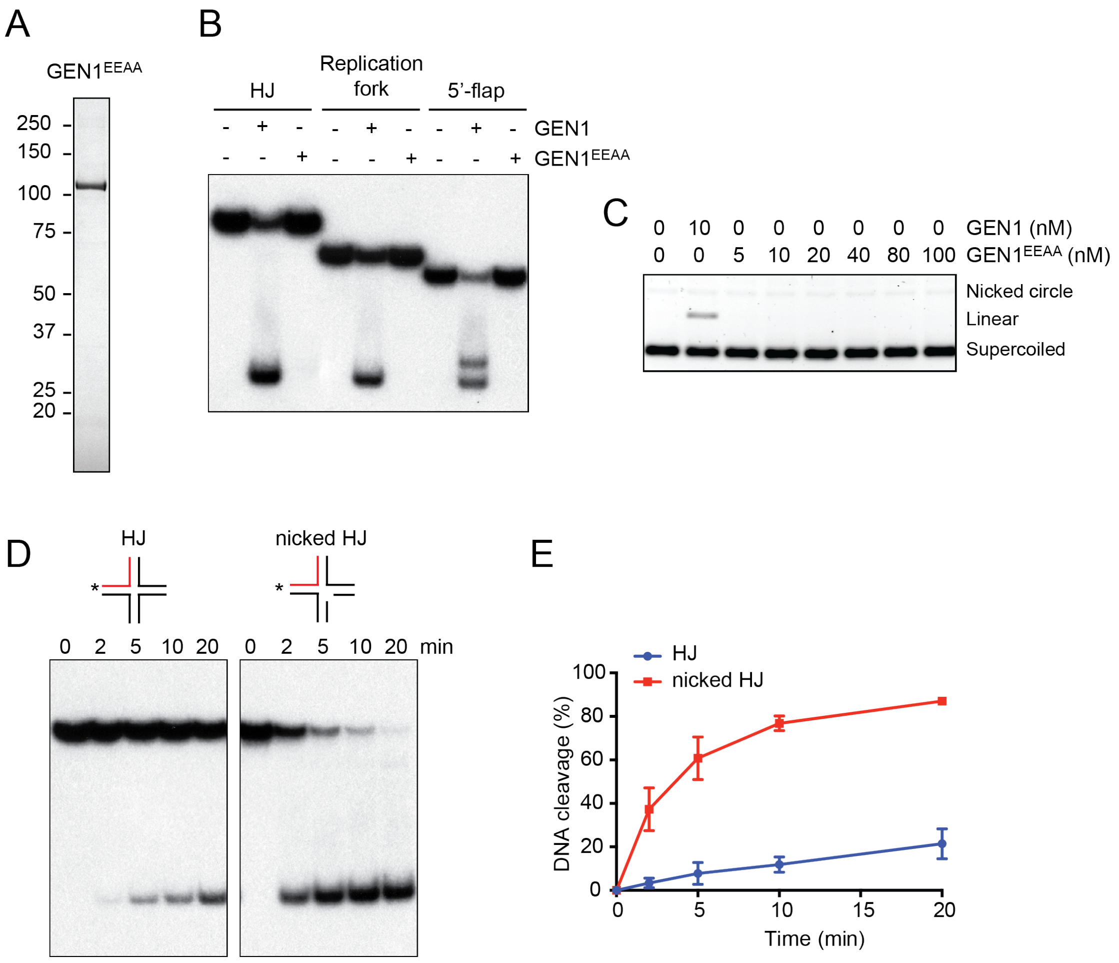

Supplementary Figure S4: Characterization of catalytically-dead GEN1 and nicked HJ cleavage by GEN1.

(A) Purified GEN1^{EEAA} was analyzed by SDS-PAGE and stained with Instant Blue. (B) The indicated DNAs (~1 nM) were incubated with GEN1 or GEN1^{EEAA} (10 nM) for 5 min. Products were analyzed by neutral PAGE. (C) Plasmid pIRbke8^{mut} (1 nM) was incubated with the indicated concentrations of GEN1 or GEN1^{EEAA} for 5 min. Products were analyzed by agarose gel electrophoresis. (D) Intact or nicked HJ X0 (50 nM) were incubated with GEN1 (5 nM) for the indicated times. Products were analyzed by neutral PAGE. (E) Quantification of DNA cleavage, as determined in (D). The data represent the mean ±s.d. of three independent experiments.

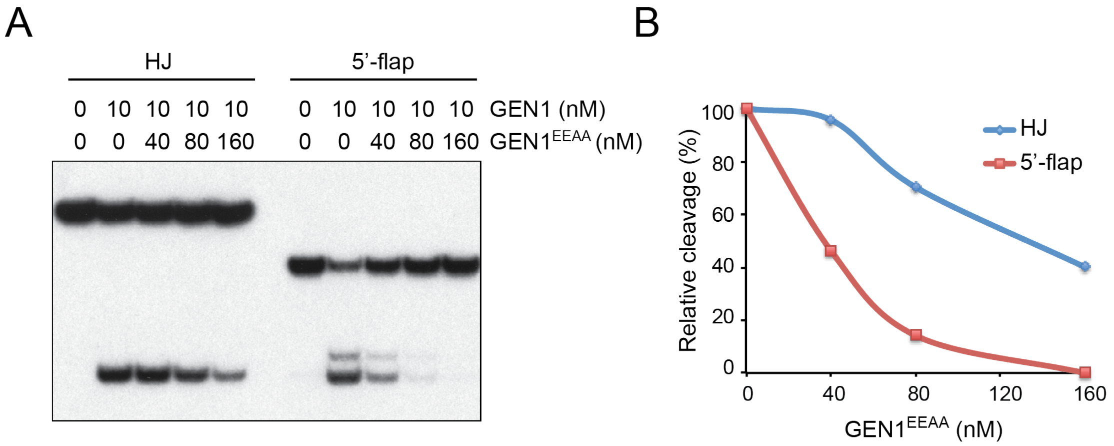

Supplementary Figure 5: Inhibition of cleavage of 5'-flap and HJ DNA mediated by catalytically-inactive GEN1.

(A) HJ or 5'-flap DNAs (20 nM) were incubated with the indicated concentrations of GEN1 and GEN1^{EEAA} for 2 min. Products were analyzed by neutral PAGE. (B) Quantification of DNA cleavage, as determined in the experiment shown in (A).

Table S1. Sequences of oligonucleotides for synthetic DNA substrates

**Oligonucleotides**	**Sequence 5' to 3'**
X0-1	ACGCTGCCGAATTCTACCAGTGCCTTGCTAGGACATCTTTGCCCACCTGCAGGTTCACCC
X0-2	GGGTGAACCTGCAGGTGGGCAAAGATGTCCATCTGTTGTAATCGTCAAGCTTTATGCCGT
X0-3	ACGGCATAAAGCTTGACGATTACAACAGATCATGGAGCTGTCTAGAGGATCCGACTATCG
X0-4	CGATAGTCGGATCCTCTAGACAGCTCCATGTAGCAAGGCACTGGTAGAATTCGGCAGCGT
X0-2(1/2)	GGGTGAACCTGCAGGTGGGCAAAGATGTCC
X0-3(1/2)	CATGGAGCTGTCTAGAGGATCCGACTATCG
X0-3(2/2)	ACGGCATAAAGCTTGACGATTACAACAGAT
X1-1T	ACGCTGCCGAATTCTACCAGTGCCTTGCTAGGACATCTTTGCCCACCTGCAGG
X1-2T	CCTGCAGGTGGGCAAAGATGTCCATCTGTTGTAATCGTCAAGCTTTATGCCGT
X1-3	ACGGCATAAAGCTTGACGATTACAACAGATGATGGAGCTGTCTAGAGGATCCGACTATCG
X1-4	CGATAGTCGGATCCTCTAGACAGCTCCATCTAGCAAGGCACTGGTAGAATTCGGCAGCGT
X26-1	GCGCTACCAGTGATCACCAATGGATTGCTAGGACATCTTTGCCCACCTGCAGGTTCACCC
X26-2	GGGTGAACCTGCAGGTGGGCAAAGATGTCCTAGCAATCCATTGTCTATGACGTCAAGCTC
X26-3	GAGCTTGACGTCATAGACAATGGATTGCTAGGACATCTTTGCCGTCTTGTCAATATCGGC
X26-4	GCCGATATTGACAAGACGGCAAAGATGTCCTAGCAATCCATTGGTGATCACTGGTAGCGC
X0-3-SP	ACGGCATAAAGCTTGACGATTACAACAGATCsATGGAGCTGTCTAGAGGATCCGACTATCG<br>(s = the SP linkage)

Duplex = X0-1 + X0-4

3'-Flap = X0-1 + X0-3(1/2) + X0-4

5'-Flap = X0-1 + X0-2(1/2) + X0-4

RF = X0-1 + X0-2(1/2) + X0-3(1/2) + X0-4

HJ X0 = X0-1 + X0-2 + X0-3 + X0-4

Asymmetric HJ X1-T = X1-1T + X1-2T + X1-3 + X1-4

HJ X26 = X26-1 + X26-2 + X26-3 + X26-4

Nicked HJ = X0-1 + X0-2 + X0-3(2/2) + *X0-3(1/2) + X0-4 (*This oligonucleotide carried a 5'-phosphate)

HJ X0-SP = X0-1 + X0-2 + X0-3 (SP) + X0-4
